# Expression of Alpha-Enolase (ENO1), Myc Promoter-Binding Protein-1 (MBP-1) and Matrix Metalloproteinases (MMP-2 and MMP-9) Reflect the Nature and Aggressiveness of Breast Tumors

**DOI:** 10.3390/ijms20163952

**Published:** 2019-08-14

**Authors:** Patrizia Cancemi, Miriam Buttacavoli, Elena Roz, Salvatore Feo

**Affiliations:** 1 Department of Biological Chemical and Pharmaceutical Sciences and Technologies (STEBICEF), University of Palermo, 90128 Palermo, Italy; 2 Centro di OncoBiologia Sperimentale (COBS), 90145 Palermo, Italy; 3 La Maddalena Hospital III Level Oncological Department, 90145 Palermo, Italy

**Keywords:** breast cancer, ENO1, MBP-1, MMP-2, MMP-9

## Abstract

Breast cancer is a complex and heterogeneous disease: Several molecular alterations cause cell proliferation and the acquisition of an invasive phenotype. Extracellular matrix (ECM) is considered essential for sustaining tumor growth and matrix metalloproteinases (MMPs) have been identified as drivers of many aspects of the tumor phenotype. Mounting evidence indicates that both α-enolase (ENO1) and Myc promoter-binding protein-1 (MBP-1) also played pivotal roles in tumorigenesis, although as antagonists. ENO1 is involved in cell growth, hypoxia tolerance and autoimmune activities besides its major role in the glycolysis pathway. On the contrary, MBP-1, an alternative product of ENO1, suppresses cell proliferation and the invasive ability of cancer cells. Since an important task in personalized medicine is to discriminate a different subtype of patients with different clinical outcomes including chances of recurrence and metastasis, we investigated the functional relationship between ENO1/MBP-1 expression and MMP-2 and MMP-9 activity levels in both tissues and sera of breast cancer patients. We focused on the clinical relevance of ENO1 and MMPs (MMP-2 and MMP-9) overexpression in breast cancer tissues: The association between the higher ENO1, MMP-2 and MMP-9 expression with a worse prognosis suggest that the elevated ENO1 and MMPs expression are promising biomarkers for breast cancer. A relationship seems to exist between MBP-1 expression and the decrease in the activity levels of MMP-9 in cancer tissues and MMP-2 in sera. Moreover, the sera of breast cancer patients grouped for MBP-1 expression differentially induced, in vitro, cell proliferation and migration. Our findings support the hypothesis of patient’s stratification based on ENO1, MBP-1 and MMPs expression. Elucidating the molecular pathways through which MBP-1 influences MMPs expression and breast cancer regression can lead to the discovery of new management strategies.

## 1. Introduction

Normal cells progressively evolve towards a neoplastic state through a multistep process, during which they acquire new traits or capability, defined as “cancer hallmarks”, including: Deregulation of cell growth and death, sustained angiogenesis, tissue invasion and metastasis. All these aspects contribute to the development of a tumorigenic and ultimately malignant, cell phenotype. Underlying these hallmarks are genome instability, tumor-promoting inflammation, reprogramming of energy metabolism and evading immune destruction [1]. Moreover, tumors represent a complex ecosystem, in which normal cells can contribute to the acquisition of hallmark traits by creating the “tumor microenvironment” [2]. New molecular insights into cancer progression pointed also to extracellular matrix (ECM) turnover, which results quantitatively and qualitatively deregulated [3,4,5]. Remarkably, increased expression and/or activity of proteolytic enzymes, especially the matrix metalloproteinases (MMPs), are frequently associated with poor prognosis in cancer patients [6]. MMP-mediated ECM remodeling can promote the release of matrix-associated growth factors, or cytokines, modulating the activity or bioavailability of signaling molecules. In addition MMP activities also result in the generation of matrix fragments displaying novel biological activity [7]. The c-MYC proto-oncogene is considered a driver oncogene, being to be involved in 20% of all human cancers. More recently, it is suggested that c-MYC could regulate as many as 15% of genes in different genomes, with more than 3.000 genes in human [8]. The pivotal role of c-MYC in regulating cell proliferation and cell death is consistent with the observations that its expression is tightly controlled by several mechanisms, including changes in transcription initiation and transcript elongation, as well as stability, turnover and translational state of mRNA [9]. The Myc promoter-binding protein-1 (MBP-1), has been identified as a protein of 37-38 kDa that binds the human c-MYC P2 promoter and negatively regulates c-MYC transcription by preventing the formation of the transcription initiation complex [8]. MBP-1 is a short form of the 48 kDa alpha-enolase protein, lacking the first 96 amino acid [9]. 

ENO1, a key glycolytic enzyme, contributes to the known “Warburg effect” that is the high glycolytic rate of cancer cells, even in presence of oxygen, and necessary to support the biosynthetic requirements of uncontrolled proliferation [10]. Moreover, the microenvironment acidification and other metabolic crosstalk alter the tumor-stroma interface, allowing the enhanced invasiveness of tumors [11,12]. It is evident that the cooperation of multiple factors rather than the involvement of a single factor is needed to induce malignancy. This hypothesis is widely considered to be more likely to define therapeutic avenues directed at multiple molecular targets for more effective cancer treatment. MBP-1 expression has been correlated with the inhibition of proliferation, migration, invasion and epithelial–mesenchymal transition (EMT) [13,14,15]. On the other hand, MMP-9 is rapidly induced and activated in response to c-MYC [16] and it was demonstrated that MBP-1 modulates MMP expression and inhibits the in vitro angiogenesis [17].

Among tumors, breast cancer is a highly heterogeneous disease, both at histological and molecular level. Patients diagnosed at the same stage of the disease and who received the same treatment, often show very different clinical responses and survival periods [18]. In this scenario, the main concern is to identify clinically homogeneous patient subgroups to evaluate the risk-benefit balance of treatment, maximizing the therapeutic efficacy, but avoiding to a significant number of subjects to be treated without receiving any benefit. 

Advances in genomics/proteomics technologies provide unprecedented opportunities for rapid advancement in translational medicine [19,20]. 

Here, we investigated the functional relationship, in a cohort of breast cancer patients, between ENO1/MBP-1 expression and MMP-2 and MMP-9 activity levels in both tissues and sera. We also analyzed in vitro the clinical relevance on predicting two important aspects of cancer: Growth and migration. Our results showed that ENO1, MMP-2 and MMP-9 are overexpressed in breast cancer tissues compared to the non-tumoral adjacent one, and how their higher expression level is associated with a worse prognosis. A relationship seems to exist between MBP-1 expression and the decrease of the activity levels of MMP-9 in tumoral tissues and MMP-2 in sera. Moreover, the sera of breast cancer patients, grouped for MBP-1 expression, differentially induced in vitro cell proliferation and migration, suggesting that both MBP-1 and MMPs reflect the nature and aggressiveness of breast tumors and can be used for patients’ stratification.

These findings support the hypothesis that MBP-1 expression can modify the tumor microenvironment decreasing the levels of molecules related to cancer progression. Further studies will be necessary to elucidate the molecular pathways through which MBP-1 influences MMPs expression and breast cancer regression, in order to translate these information into the clinical practice.

## 2. Results

### 2.1. Overexpression of ENO1 in Breast Cancer Tissues 

**Figure 1:**
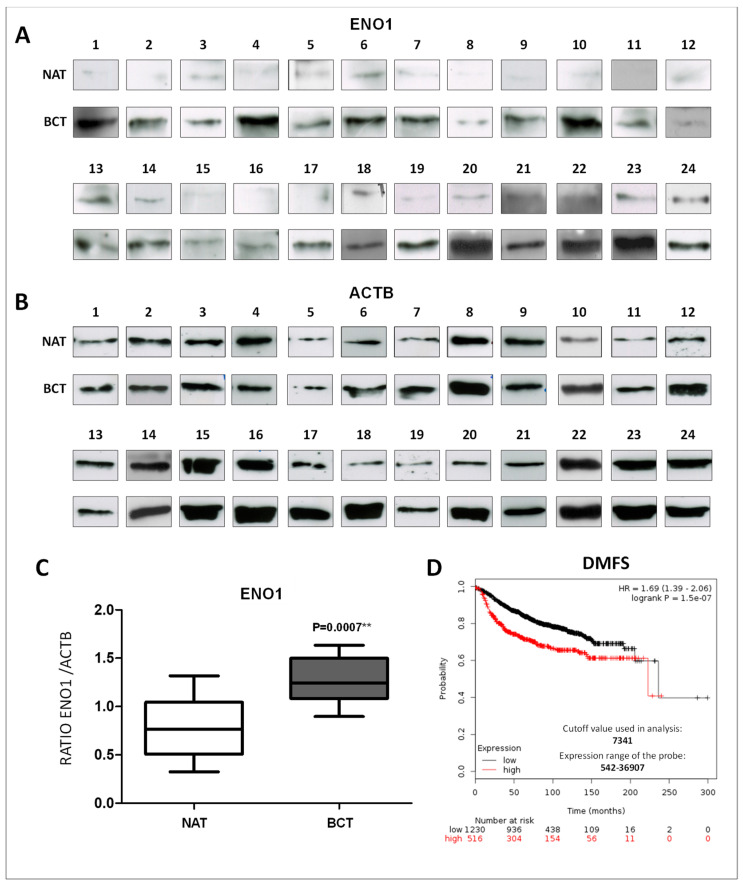
ENO1 expression between normal and breast cancer tissues and prognostic significance. (**A**,**B**) Western blot analysis of ENO1 and actin beta (ACTB) using total lysates from breast tumors (BCT) and paired non-tumoral adjacent tissues (NAT); *n* = 24. (**C**) Graphic quantification of western blot results. Each data point is the average of three independent experiments. Error bars represent the standard deviation and the *p* value indicates statistical significance. (**D**) Survival analysis in breast cancer patients obtained from the Kaplain–Meir plotter database relative to ENO1 mRNA expression.

The expression levels of ENO1 (48 kDa) was evaluated by western blot in a subset of 24 breast cancer tissues and their paired non-tumoral adjacent tissues. Protein loading was ascertained by actin beta (ACTB) expression (Figure 1A). All non-tumoral adjacent tissues showed very low expression of alpha-enolase, whereas its high expression was observed in the paired tumor samples. Statistical analysis of Western blot data confirmed the presence of significantly higher levels ofENO1 in breast cancer samples rather than in normal tissues (Figure 1B). The prognostic value of ENO1 mRNA expression in breast cancer patients using the Kaplan–Meier plotter database was also investigated. Patients were split into two groups by using the best cut-off of probe expression, and survival was evaluated as distant metastasis-free survival (DMFS). Results showed that higher ENO1 expressions were significantly associated with a worse prognosis (highlighted in red, Figure 1C; *p* = 1.5 × 10^−7^). Collectively, these results suggest that ENO1 is overexpressed both at the mRNA and the protein level in breast cancer tissues, and the upregulated ENO1 mRNA is correlated with a worse prognosis.

### 2.2. Upregulation of MMP-9 and MMP-2 in Breast Cancer Tissues 

**Figure 2:**
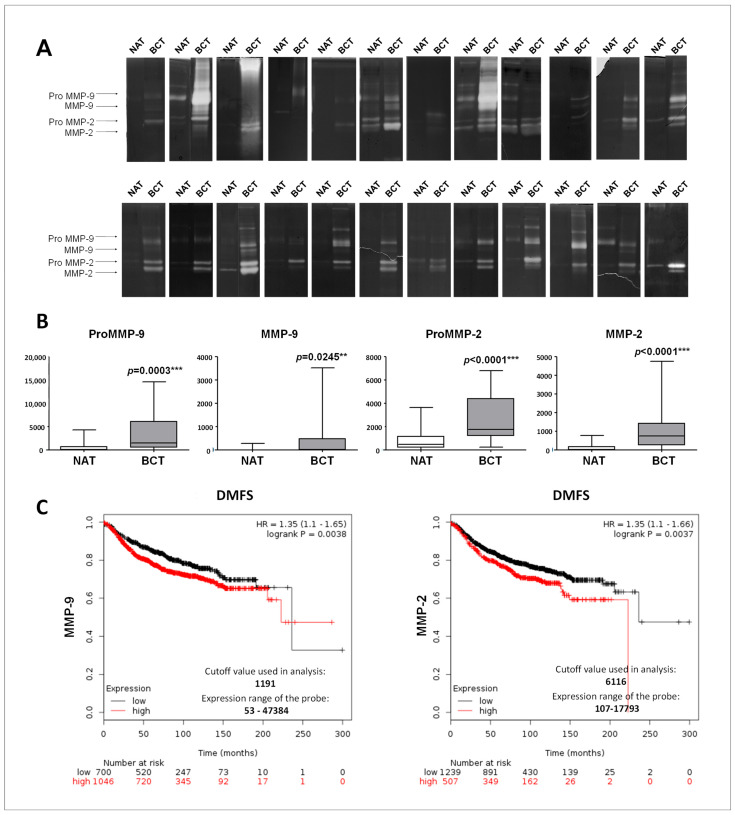
MMP-9 and MMP-2 enzymatic activity in normal and breast cancer tissues and prognostic significance. (**A**) Gelatin zymography was performed using total lysates from breast tumors (BCT) and paired non-tumoral adjacent tissues (NAT); *n* = 24. (**B**) Densitometric analysis of the gelatinolytic bands. Each data point is the average of three independent experiments. Error bars represent standard deviation and *p* values indicate statistical significance. (**C**) Survival analysis in breast cancer patients obtained from Kaplain–Meir plotter database relative to MMP-9 and MMP-2 expression.

The proteolytic degradation of the ECM operated by MMPs represent a key aspect of tumor progression, since ECM is the first barrier against cell invasion. Therefore, in the same subset of 24 patients, the enzymatic activities of MMP-2 and MMP-9 were also evaluated by zymography. As shown in Figure 2A, the majority of the breast cancer tissues were positive for the lytic activities corresponding to the latent and activated form of MMP-2 and MMP-9. On the contrary, the non-tumoral counterparts were generally positive only for the latent form of MMP-2 and MMP-9. Further, lytic bands of high molecular weight, corresponding to the homodimeric forms of MMP-9 and complexes of pro-MMP-9/TIMP-1, respectively, also occurred in some patients, as already reported [21,22]. The densitometric quantification of the activity levels was performed as relative volumes, calculated as an area X optical density, using ImageJ software. As shown in the graphs (Figure 2B), the activity levels of pro-MMP-2, MMP-2, pro-MMP-9, and MMP-9 were significantly higher in the tumoral tissues, as compared to the non-tumoral adjacent tissues. Overexpression of MMP-2 and MMP-9 mRNA were also correlated with poor outcome as evaluated by distant metastasis free survival (DMFS) in the Kaplan–Meier plotter database (Figure 2C). Collectively, these results suggest that MMPs are overexpressed in breast cancer tissues, and the upregulated MMP-9 and MMP-2 mRNAs are correlated with a worse prognosis.

### 2.3. MBP-1 Expression in Breast Cancer Tissues

**Figure 3:**
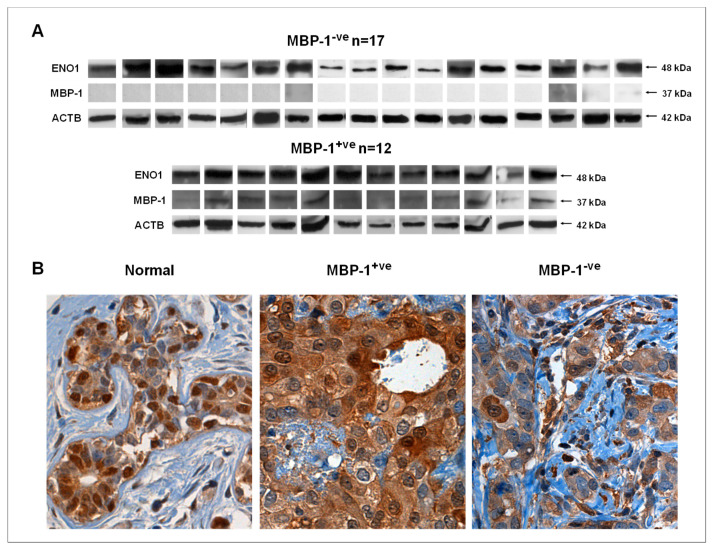
Myc promoter-binding protein-1 (MBP-1) expression in breast cancer tissues. (**A**) Western blot detection of ENO1 and MBP-1 in tumor tissues from 29 from breast cancer patients; (**B**) representative immunohistochemical staining on sections of a healthy mammary tissue and two breast cancer tissues, respectively positive and negative for MBP-1 expression. Cytoplasmic staining is referred to ENO1 while nuclear staining is attributable to MBP-1 expression (Magnification 400×).

The concomitant expression of ENO1 (48 kDa) and MBP-1 variant (37 kDa) was evaluated in a cohort of 29 breast cancer tissues. Protein loading was ascertained by ACTB expression. Among the analyzed patients, 12 were positive for MBP-1 expression while 17 were negative (Figure 3A). Moreover, in all cases, the expression levels of ENO1 are higher than MBP-1. Western blot analysis results were also validated by immunohistochemistry by using the monoclonal antibody Eno 19/8 which recognizes an epitope within the aminoacids residues 275-344 [23,24]. As previously reported, cytoplasmatic staining is due to the ENO1 expression, while nuclear staining depends on the MBP-1 expression. A panel of representative immunohistochemical (IHC) staining images of MBP-1 in normal mammary and breast cancer tissues (both positive and negative) were shown in Figure 3B. IHC analysis revealed a strong MBP-1 staining in nuclei of healthy mammary tissues, while in breast cancer tissues only the selected samples showed the nuclear staining. In all cases, cytoplasmic staining correspond to ENO1 (48 kDa) expression. According to the western blot and immunohistochemical analyses on MBP-1 expression, breast tumors were classified as MBP-1^−ve^ and MBP-1^+ve^. 

### 2.4. MBP-1 Expression is Associated with MMPs Down-Regulation

**Figure 4:**
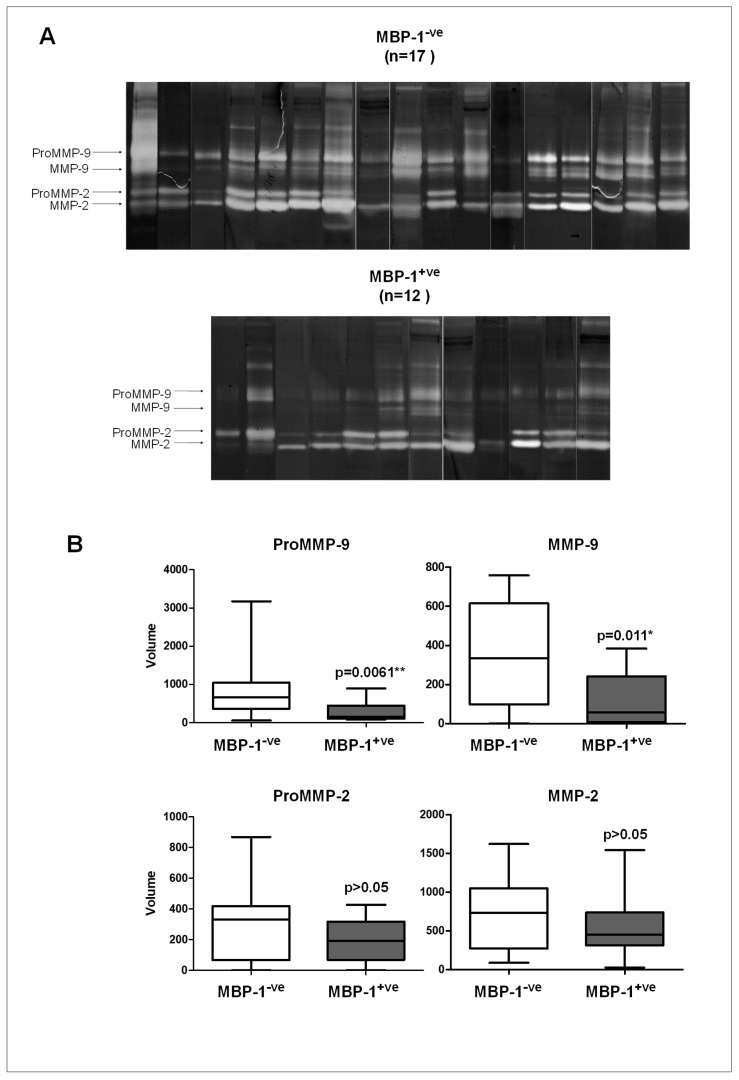
Matrix metalloproteinases (MMP)-9 and MMP-2 activity levels in breast cancer tissues according to MBP-1 expression. (**A**) Gelatin zymography of 29 breast cancer patients split into two groups (MBP-1^-ve^ and MBP-1^+ve^) according to the MBP-1 expression. (**B**) Quantification of lytic bands. Each data point is the average of three independent experiments. Error bars represent standard deviation and *p* values indicate statistical significance.

To verify if the expression of MMPs in breast cancer tissues was different between MBP-1^-ve^ and MBP-1^+ve^ tumors, zymographic analysis in the two groups of patients was performed (Figure 4A). The obtained results clearly indicated a significant down regulation of MMP-9 (both the proenzyme and the active enzyme) in MBP-1^+ve^ tumors (Figure 4B). MMP-2 activity levels were not significantly affected. 

**Figure 5:**
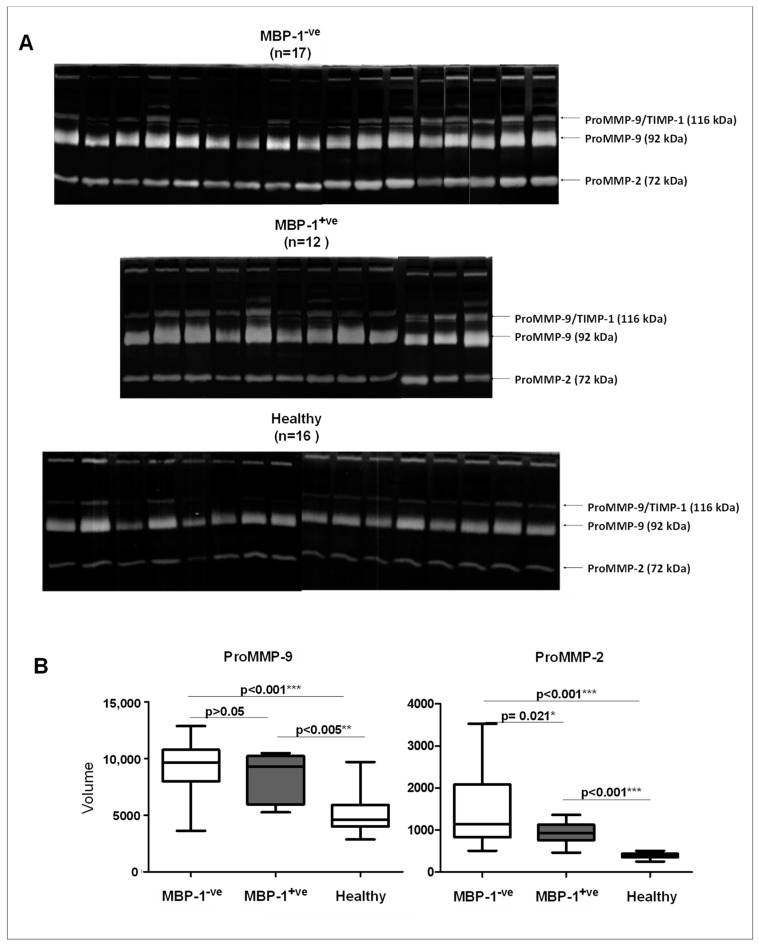
ProMMP-9 and ProMMP-2 activity levels in breast cancer sera according to MBP-1 expression and healthy subjects. (**A**) Gelatin zymography of sera of MBP-1^-ve^, MBP-1^+ve^ breast cancer patients and healthy subjects; (**B**) quantification of lytic bands. Each data point is the average of three independent experiments. Error bars represent standard deviation and *p* values indicate statistical significance.

Following this, to evaluate if the MMPs activity levels could be also affected in sera samples of breast patients previously stratified as MBP-1^-ve^ and MBP-1^+ve^, the zympgraphic analysis on sera samples collected before surgery was performed. Sera samples (*n* = 16) of healthy donors were used as control specimens. In all the analyzed sera, only the enzymatic activity of ProMMP-9 and ProMMP-2 was detected, while no enzymatic activity was detected for the activated forms (Figure 5A). Densitometric analysis of the lytic bands showed that the activity values of pro-MMP-2 were significantly higher in MBP-1^-ve^ breast cancer sera compared to MBP-1^+ve^ and healthy sera (Figure 5B). No statistical differences were found among the three groups for ProMMP-9 activity.

### 2.5. Contributing Role of MBP-1^-ve^ and MBP-1^+ve^ Breast Cancer Sera in Cell Proliferation and Aggressiveness

**Figure 6:**
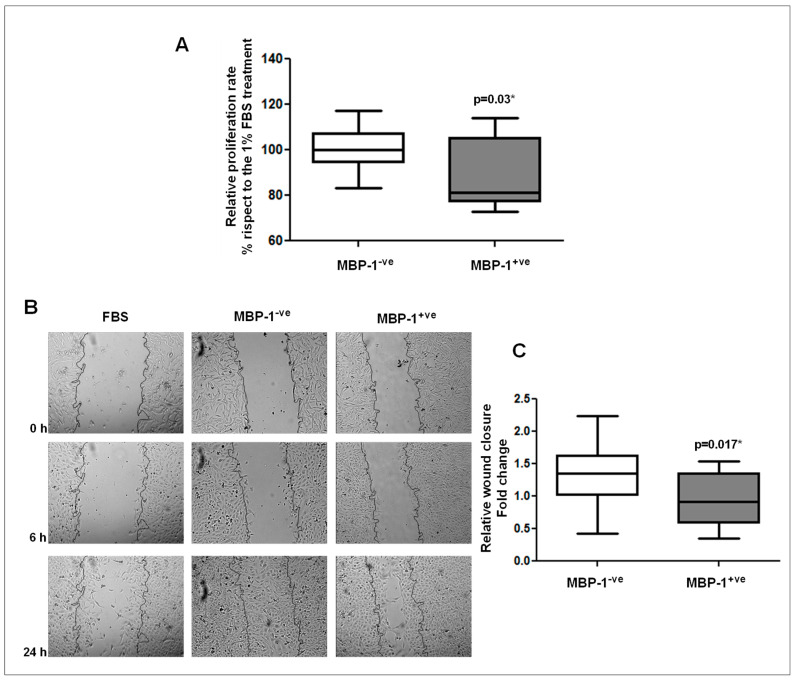
Effect of MBP-1^−ve^ and MBP-1^+ve^ breast cancer sera on cancer cell proliferation and migration. (**A**) Relative cell growth measured by MTT assay. MDA-MB-231 cells were starved for 24 h (0% FBS) and then treated for 24 h with 1% FBS or 1% of human sera from breast cancer patients, previously grouped as MBP-1^−ve^ and MBP-1^+ve^. (**B**) Wound healing of MDA-MB-231 cells after stimulation with 1% of FBS and 1% of human sera from breast cancer patients, previously grouped as MBP-1^-ve^ and MBP-1^+ve^. Migration at the edge of the scratch was analyzed at 0, 6 and 24 h (magnification, 100×). (**C**) Quantification of cell migration was performed measuring the scratch wound, and results are expressed relative to the closure of 1% FBS treatment.

To establish if the sera of MBP-1^-ve^ and MBP-1^+ve^ breast tumors were able to affect cell growth and migration, the MDA-MB-231 cells were used as prototype of highly proliferative and metastatic tumor model. Cell proliferation was evaluated by MTT assay after 24 h of treatment with 1% sera from MBP-1^-ve^ or MBP-1^+ve^ breast cancer patients, compared to 1% Foetal Bovin Serum (FBS) treatment. The treatment with MBP-1^+ve^ breast sera reduced significantly cell proliferation rate, although a high heterogeneity was recorded with some sera (Figure 6A). The effect of MBP-1^-ve^ and MBP-1^+ve^ breast sera on MDA-MB-231 cell motility was also evaluated by in vitro wound healing assay. As shown in Figure 6B, the migratory capability of MDA-MB-231 cells treated with MBP-1^-ve^ sera was considerably increased compared to the FBS treatment. Interestingly, the enhanced migration induced by MBP-1^−ve^ sera was diminished in MDA-MB-231 cells treated with MBP-1^+ve^ sera (Figure 6B,C). In summary, the obtained results suggest that MBP-1^+ve^ tumors are less aggressive than MBP-1^-ve^ tumors.

## 3. Discussion

Although several efforts have been directed toward understanding the molecular mechanisms underlying tumorigenesis, the prognosis of different patients is much more difficult to define based on some molecular/clinical parameters. The demand for novel/personalized therapies is continuously increasing, therefore it is urgent to find new biomarkers for better classification and prognosis prediction, as well as the response to therapy.

Here, we provide new evidences about the clinical relevance of ENO1 and MMPs (MMP-2 and MMP-9) overexpression in breast cancer tissues. All non-tumoral breast tissues showed low expression/activity of both ENO1 and MMP-2 and MMP-9, whereas higher expression/activity was observed in the paired tumour samples. A clear association between the higher mRNA expression level of ENO1, MMP-2 and MMP-9 with a worse prognosis was already detected, suggesting that the elevated ENO1 and MMPs are promising biomarkers for breast cancer. 

ENO1 is a glycolytic enzyme and a multifunctional protein that plays a crucial role in a variety of biological and pathophysiological processes [25]. According to the well-known Warburg effect, cancer cells highly express almost all glycolytic enzymes [26], including ENO1, which is upregulated both at the mRNA and the protein level in several tumors including breast [27]. Aberrant expression of ENO1 has been associated with multiple tumor progression both in vitro [28,29,30,31] and in vivo [32,33,34,35,36] and higher levels of ENO1 has been correlated with poor prognosis [37,38]. This evidence can be partially explained considering the Warburg effect [30], consisting in the ability to switch the cell metabolism from predominantly oxidative to glycolysis and the production of lactate, even if the oxygen is plentiful. Switching to the aerobic glycolysis is a key characteristic of cancer metabolism, being critical for both tumor cell growth and migration [39]. In fact, the acidification of extracellular milieu providing a favorable microenvironment for the activation of proteases, e.g., MMPs, induce ECM degradation and facilitate tumor cells to metastasize [40]. MMPs, originally described as regulators of ECM remodeling and facilitators of tumor growth, modulate the activity of both inducers and inhibitors of tumorigenesis, by processing cytokines, angiogenic and growth factors, and their respective receptors, as well as altering cell adhesion and stimulating epithelial to mesenchymal transition [41,42]. The expression and activity of MMPs are increased in almost every type of human cancer, including breast cancer, and correlate with an advanced tumor stage, increased invasion, metastasis, and shortened survival [43,44]. Generally, in tumors, MMPs are deregulated by transcriptional changes rather than genetic alterations such as amplification or activating mutations. This might be the result of activation of oncogenes or loss of tumor suppressors. MBP-1, a nuclear short variant of ENO1 [9] acting as a transcriptional repressor, could represent a good link for MMPs regulation. MBP-1 is an oncosuppressor, negatively regulating cell proliferation or promoting cancer cell apoptosis when overexpressed in vitro [45,46,47]. Its tumor-suppressor function depends on the repression of at least three gene targets, namely: c-MYC, COX2 and ERBB2 [9,48,49]. The MBP-1-mediated repression also depends on the association with different partners, such as: MIP-2/sedlin [50], histone deacetylase 1 [48,51], the kelch protein NS1-BP [23], and the intracellular domain of the Notch 1 receptor [52]. Moreover, MBP-1 suppresses, in vitro, tumor invasion and metastasis in different models of cancer [13,49,53]. In breast cancer, MBP-1 expression inversely correlates with the ErbB2 and Ki67 expression levels and it is a good predictor of disease-free survival [24]. 

To our knowledge this is the first study reporting the association of MBP-1 expression and MMPs activity in clinical breast cancer tissues. In particular, our analysis showed a significant lower activity of both ProMMP-9 and MMP-9 in MBP-1^+ve^ patients. In fact, MMP-9 is induced under conditions that require tissue remodeling (including tumor invasion) [54]. A direct link has been established between MMPs transcriptional regulation by COX-2 [55], HER2 [56] and c-MYC [57,58], therefore we hypothesize that MBP-1 expression, through the downregulation of its specific targets, can regulate the tissue levels of MMP-9. 

Our analysis showed a relationship between MBP-1 expression and the decrease of ProMMP-2 in breast cancer sera, proving for the first time that MBP-1 tissue expression could influence tumor aggressiveness by influencing the composition of the microenvironment and/or the mobilization of factors related to the biological features of the tumor. More interestingly, MBP-1 expression influences MMPs activity levels in different compartments (ProMMP-9 and MMP-9 in breast tissues and ProMMP-2 in sera) underlining unique functions for both proteolytic enzymes. We previously showed the complexity of the interactive networks (partly common and partly exclusive) through which MMP-2 and MMP-9 drive their functions [59], which also depend on the cell type and environment. These complex interactive molecular circuits suggest their potential involvement in other important cellular activities, besides that of remodeling the extracellular matrix. 

Because cancer aggressiveness depends also on the ability to induce proliferation and migration we further examined the effects of sera from MBP-1^-ve^ patients versus MBP-1^+ve^ patients to stimulate proliferation and migration in the MDA-MB-231 breast cancer cells. The cell proliferation rate and migration was significantly reduced by using the MBP-1^+ve^ compared to MBP-1^-ve^ breast cancer sera, although a high heterogeneity was recorded with some sera. The modulation of cell proliferation and migration accounts for specific factors that are differentially enriched in each serum, but collectively this is the first indication that breast tumors MBP-1^+ve^ have a lower proliferative and migratory capacity.

Our results are in agreement with the observation that MBP-1 expression results in the modulation of MMP-2 expression [60], the inhibition of in vitro angiogenesis and the regression of primary [53] and metastatic breast tumor growth [13]. Endogenous levels of ENO1 are higher than MBP-1 due to differences in translational efficiency, translational regulation and posttranslational stability [61]. Although the mechanisms regulating MBP-1/ENO1 ratio are not fully understood, several factors such as hypoxia [62], Endoplasmic Reticulum stress [63] and glucose concentrations [64] were described to influence MBP-1 and ENO1 transcription, strongly suggesting that posttranscriptional mechanisms might play a role in the regulation of MBP-1 expression in breast cancer cells. Accordingly, a molecular switch of ENO1 translation in favor of MBP-1 translation could convert very aggressive breast cancer tumors into less aggressive tumors.

## 4. Materials and Methods

### 4.1. Patients, Tissue and Sera Samples 

A total of 53 patients diagnosed with breast ductal infiltrating carcinomas with histological grading G2/G3 and without clinically apparent metastases were involved in this study. The patients did not receive any cytotoxic/endocrine treatment prior to surgery. The tissue samples were obtained following surgical interventions at “La Maddalena” Hospital of Palermo and were immediately cryo-preserved at −80 °C until use, as already reported [65,66,67]. The non-tumoral adjacent tissues were located at least 5 cm away from the primary tumor. Blood samples were obtained prior to surgery, using plastic tubes without coagulation accelerators to prevent the release of gelatinases during platelet activation. The study was carried out after fulfilling all required ethical standards with the informed consent of patients and with the approval of the Institutional Review Board (N°515/2008, 13 May 2008) from the La Maddalena Hospital. Healthy sera (*n* = 16) were taken from healthy volunteers.

### 4.2. Tissues and Sera Processing 

The frozen tissues were homogenized in an ice bath with 50 mM Tris-HCl pH 7.5, 0.003% penicillin, 0.005% streptomycin and incubated under rotation overnight at 4 °C. Tissue lysates were centrifuged several times at 10,000 rpm for 20 min to remove cell debris. Protein content was quantified by Bradford assay, as already reported [68,69]. Blood, 30 m after collection, was centrifuged at 1600× *g* for 10 min, and sera were aliquoted and used only once to prevent enzymatic activation due to freeze-thawing processes.

### 4.3. Western Blotting Analysis 

Aliquots containing 20 µg of cell lysates from breast cancer tissues and paired non tumoral adjacent tissues were subjected to SDS polyacrylamide gel electrophoresis and transferred into a nitrocellulose membrane (HyBond ECL, Amersham, GE Healthcare Bjorkgatan, Uppsala, Sweden). Membranes were blocked with 5% milk in T-TBS solution for 1 h at room temperature and then incubated overnight at 4 °C with a mouse monoclonal antibody for ENO1 or Actin β by Santa Cruz Biotechnology (Santa Cruz, CA, USA). A monoclonal specific antibody to the C-terminus (ENO19/8) was affinity-purified on ProA-Sepharose 4B, as previously described [24]. Following incubation with the mouse peroxidase-linked antibody, the reaction was revealed by the ECL detection system, using high performance films (Hyperfilm ECL, Amersham, GE Healthcare Bjorkgatan, Uppsala, Sweden), as already described [70,71,72]. The correct protein loading was ascertained by red Ponceau staining and immunoblotting for Actin *β*. Densitometric analysis was performed with ImageJ software to quantify signals. When the activated forms migrated as multiple bands in consequence of post-translational modifications, the quantification was performed considering all bands, defining a single region of interest.

### 4.4. Kaplan–Meier Plotter Database Analysis

The KM Plotter database [73] (http://kmplot.com/analysis/), able to assess the effect of 54,675 genes on survival using 10,461 cancer samples, including 5143 breast, was queried to evaluate the prognostic values of ENO1, MMP-2 and MMP-9 in breast cancer. The desired probes ID was entered into the database and patients were split into high and low expression group by the best cut-off values of mRNA expression. When the best cut-off is selected, all possible cut-off values between the lower and upper quartiles are computed, and the best performing threshold is used. 

### 4.5. Gelatin Zymography 

Gelatin zymography is used to detect gelatinases activity (MMP-2 and MMP-9) after SDS-PAGE electrophoresis separation. MMP-2 and MMP-9 remain inactive while they are with their pro-domains. They need proteolytic processing or denaturation to get activated. Denaturation induced by SDS in SDS-PAGE, activate the enzymes. MMP-2 (72 kDa) and MMP-9 (92 kDa) can be detected on gelatin zymograms as two-three white bands (pro and active forms) after staining with Coomassie Blue. Aliquots containing 18 µg of cell lysates from breast cancer tissues and paired non tumoral adjacent tissues or 10 µl of sera previously diluted 1:25 were separated by electrophoresis on a 7.5% sodium dodecyl sulfate (SDS)–polyacrylamide gel containing 0.1% gelatin, under non-reducing conditions [4,74]. After electrophoresis, the gels were washed for 1 h with a buffer containing 50 mM Tris-HCl, pH 7.5 and 2.5% Triton X-100 to remove SDS and then incubated for 18 h at 37 °C with the activation buffer composed of 50 mM Tris-HCl pH 7.5, 150 mM NaCl, 5 mM CaCl_2_. Gels were stained with Coomassie Brilliant Blue G 250 and de-stained with H2O milliQ. The bands intensity was measured with ImageJ software.

### 4.6. Immunohistochemistry 

Immunommunohistochemistry was performed on tissue sections from archived formalin-fixed, paraffin-embedded tissue blocks from patients. Sections of 3 µm were cut, deparaffinized in xylene and hydrated in a graded series of alcohol. After antigen retrieval in citrate buffer, staining was performed using the BenchMark automated staining system (Ventana Medical System, Tucson, AZ, USA) with primary antibodies against ENO1 affinity purified monoclonal antibodies (mAbs) (ENO19/8 1.0 µg/mL). Rabbit anti-human polyclonal antibodies were used as a negative control (dilution 1:500). ENO1 expression into the cytoplasm and nucleus was empirically determined. In addition, for nuclear staining, the percentage of cells labeling was graded as negative for ≤20%, or positive for >20%. 

### 4.7. Cell Cultures

MDA-MB-231 cells were cultured in DMEM medium (Gibco, Paisley, UK) supplemented with 10% heat-inactivated fetal bovine serum and 100 U/mL penicillin, 100 μg/mL streptomycin and then maintained at 37 °C and 5% CO_2_, as already described elsewhere [4,5,75]. 

### 4.8. Cell Proliferation Assay

The cell proliferation rate was determined using MTT assay (Promega, Madison, WI, USA). Briefly, cells were plated at 5 × 10^3^ cells/well in 96-well plates. After adhesion achievement, cells were starved for 24 h without serum and then treated for 24 h with 1% of FBS or 1% of breast cancer sera of MBP-1^-ve^ and MBP-1^+ve^ tumors. An amount of 20 μL of the cell titer 96^®^AQ_ueous_ reagent was added to each well after three washes with phosphate buffer saline (PBS) and incubated for 14 h at 37 °C in a CO_2_ incubator. The absorbance was recorded at 490 nm using a 96-well plate reader (Spark^®^ 20M, Tecan Trading AG, Switzerland). The percentage of cell viability was calculated with respect to untreated control cells for each treatment after subtraction of the blank. 

### 4.9. In Vitro Scratch Assay 

Scratch assay was performed to analyze cell migration after FBS or breast cancer sera stimulation. Cells were seeded on 24-well plates and grown to 100% confluence. Wounds were created by scraping the monolayer of cells with a sterile pipette tip, washed with PBS to remove the floating cells and then incubated with fresh medium in the presence of 1% of FBS or 1% of breast cancer sera of MBP-1^-ve^ and MBP-1^+ve^ tumors. The images of scratched area were captured (at 40× magnification) using an inverted microscope equipped with digital camera immediately after wounding and after 6 and 24 h. The scratched area was calculated by ImageJ software. 

### 4.10. Statistical Analysis

All data were analyzed by using GraphPad Prism version 5.0 for Windows (GraphPad Software, Inc. La Jolla, CA, USA) and presented as means ± standard deviation of at least three experiments. The statistical significance of differences was assessed by Mann Whitney or the Kruskal–Wallis test. * *p* < 0.05 was considered significant; ** *p*< 0.01 highly significant and *** *p* < 0.001 very highly significant. The data in the graphs are expressed as median ± SD.

## 5. Conclusions

Our data, in addition to previous evidence allow a better breast patients stratification based on homogeneous prognosis, in fact while the high expression of ENO1 and MMPs are associated with a worse prognosis, the expression of MBP-1 is associated with a decrease in MMPs activity, a less aggressive biological features of the tumor and better prognosis.
