# Correction: Cancemi et al. Expression of Alpha-Enolase (ENO1), Myc Promoter-Binding Protein-1 (MBP-1) and Matrix Metalloproteinases (MMP-2 and MMP-9) Reflect the Nature and Aggressiveness of Breast Tumors. *Int. J. Mol. Sci.* 2019, *20*, 3952

**DOI:** 10.3390/ijms27021085

**Published:** 2026-01-22

**Authors:** Patrizia Cancemi, Miriam Buttacavoli, Elena Roz, Salvatore Feo

**Affiliations:** 1 Department of Biological Chemical and Pharmaceutical Sciences and Technologies (STEBICEF), University of Palermo, 90128 Palermo, Italy; miriam.buttacavoli@unipa.it (M.B.); salvatore.feo@unipa.it (S.F.); 2 Centro di OncoBiologia Sperimentale (COBS), 90145 Palermo, Italy; 3 La Maddalena Hospital III Level Oncological Department, 90145 Palermo, Italy; roz@lamaddalenanet.it

In the original publication [1], Figure 2A has been updated to replace an inadvertent duplication from the original Figure 2A. The error occurred for the zymography of a sample patient, due to the cropping of the corresponding image in the original gel. The corrected Figure 2A appears below. The authors state that the scientific conclusions are unaffected. This correction was approved by the Academic Editor. The original publication has also been updated.

## Figures and Tables

**Figure 2:**
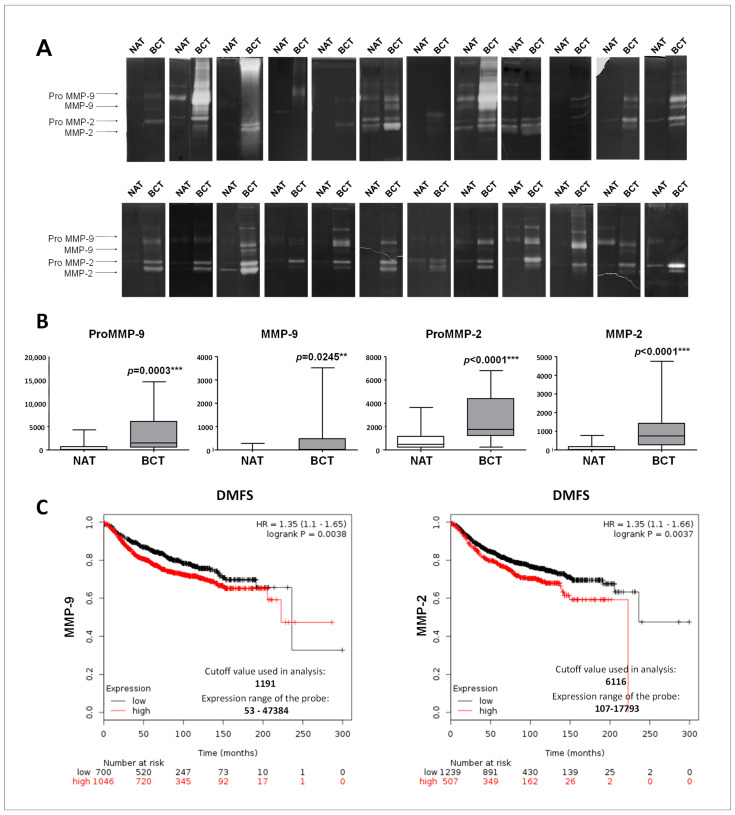
MMP-9 and MMP-2 enzymatic activity in normal and breast cancer tissues and prognostic significance. (**A**) Gelatin zymography was performed using total lysates from breast tumors (BCT) and paired non-tumoral adjacent tissues (NAT); *n* = 24. (**B**) Densitometric analysis of the gelatinolytic bands. Each data point is the average of three independent experiments. Error bars represent standard deviation and *p* values indicate statistical significance. (**C**) Survival analysis in breast cancer patients obtained from Kaplain–Meir plotter database relative to MMP-9 and MMP-2 expression.
